# Genome sequence of *Propionimicrobium lymphophilum* ARP01 isolated from the human vaginal tract

**DOI:** 10.1128/mra.00178-26

**Published:** 2026-06-24

**Authors:** Audrey R. Peel, Helen Churchill, Matthew R. Hayward, Brian A. Klein

**Affiliations:** 1 Microbiome & Hygiene, dsm-firmenich, Lexington, Massachusetts, USA; 2 Department of Biology, Tufts University 1810, Medford, Massachusetts, USA; University of Wisconsin-Madison, Madison, Wisconsin, USA

**Keywords:** *Propionimicrobium*, Nanopore sequencing, vaginal microbiota

## Abstract

*Propionimicrobium lymphophilum* strain ARP01 was isolated from a vaginal swab taken from a healthy 52-year-old Caucasian woman based in the United States. Whole-genome sequencing was performed on strain ARP01 using Oxford Nanopore Technologies. The genome assembly yielded a single contig of 2.2 Mbp.

## ANNOUNCEMENT

*Propionimicrobium lymphophilum* was first described by John Torrey in 1916 from human lymph gland isolates, and he named the species *Bacillus lymphophilus* (1). It was reclassified in 1972 as *Propionibacterium lymphophilum* by Johnson and Cummins, and again in 2002 by Stackebrandt et al. when the species was brought to its current name of *Propionimicrobium lymphophilum* (2, 3).

*P. lymphophilum* is commonly found in urine samples (4). Several studies have shown that *P. lymphophilum* can cause urinary tract infections, as well as monosymptomatic nocturnal enuresis (5, 6). Additionally, *P. lymphophilum* has been linked to two reported cases of bacteremia in elderly patients (7, 8). *P. lymphophilum* has also been isolated from the human vaginal tract (9, 10).

*P. lymphophilum* may play a role in the development of prostate cancer through steroid biosynthesis (11) as the species possesses genes that encode an enzyme capable of modifying cortisol, leading to the creation of 11-oxy-androgen (12), a steroid linked to prostate cancer (13).

*P. lymphophilum* strain ARP01 was isolated from a vaginal swab provided by a healthy 52-year-old woman from the United States (Medix Biochemica). The swab was thawed and then vortexed in 1 mL of 1× PBS for 1 min. The solution was plated onto YCFAC+B agar (Anaerobe Systems) and incubated anaerobically for 4 days at 37°C. After incubation, a single colony was selected and resuspended in PYG broth (Anaerobe Systems), and then grown anaerobically for 48 h at 37°C. Following broth incubation, the culture was split for storage in a PBS/glycerol cryoprotectant solution at −80°C and pelleting for DNA extraction and sequencing. Both initial identification and subsequent confirmation were done with whole-genome sequencing.

DNA was extracted using a QIAamp PowerFecal Pro DNA QIAcube Kit on a QIAcube HT, per manufacturer’s instructions (Qiagen). Extracted DNA concentration and quality were confirmed using a Qubit High Sensitivity dsDNA Kit (Thermo Fisher Scientific). The sequencing library was prepared following the Oxford Nanopore Rapid Barcoding 96 kit version 14, SQK-RBK114.96 (Oxford Nanopore Technologies) and was sequenced using an Oxford Nanopore Technologies GridION X5, flow cell type R10.4.1. Reads were base-called using the Super Accuracy Analysis setting (MinKNOW 24.02.16, Dorado 7.3.11), resulting in 29,205 reads with an N50 length of 4,208 bp (Table 1).

**TABLE 1:** *Propionimicrobium lymphophilum* ARP01 information and genome metrics

Sample name	Species	Assembly data				GenBank accession no.	No. CDSs	Coverage (×)	GC content %	No. tRNAs	16S copies	Culture conditions
		No. reads	No. contigs	N50 (bp)	Size (bp)							
ARP01	*Propionimicrobium lymphophilum*	29,205	1	2,226,193	2,226,193	CM145505.1	2,049	31	55.47	45	2	PYG, anaerobic

**Fig 1:**
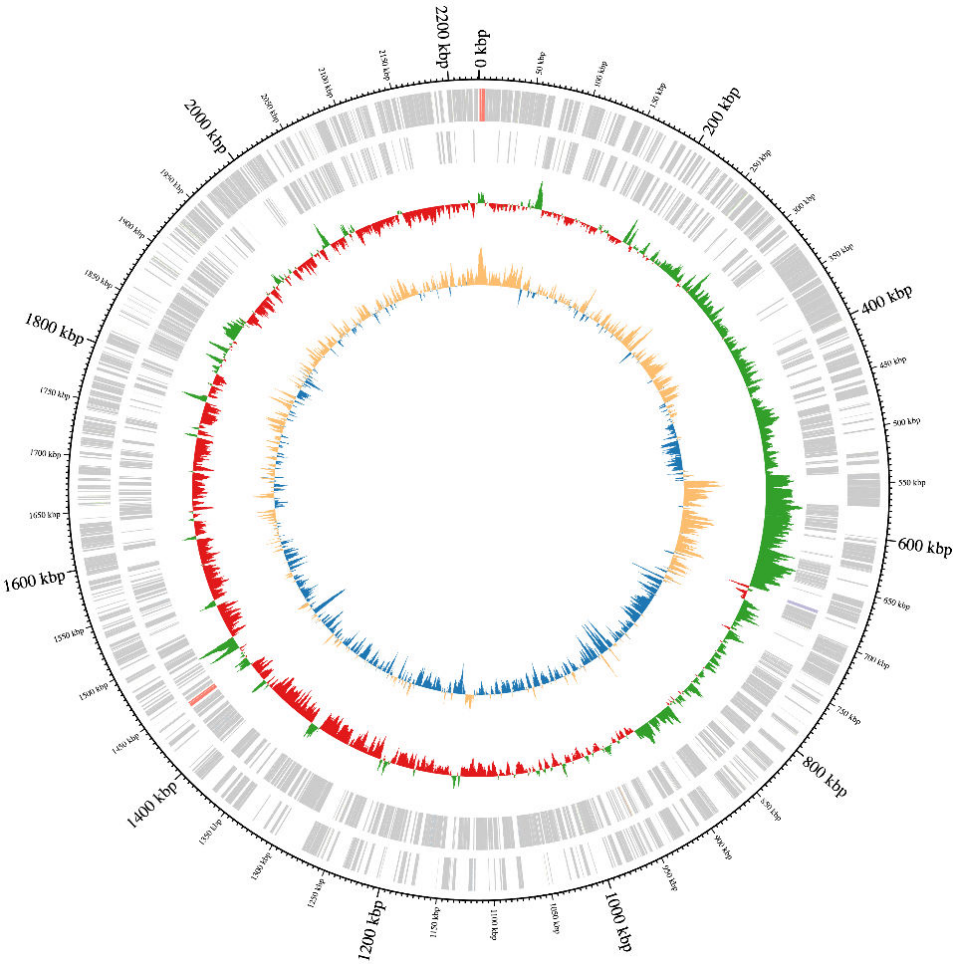
*Propionimicrobium lymphophilum* ARP01 genome visualized using Bakta (Galaxy version 1.9.4+galaxy1).

The genome was assembled on Galaxy; default parameters were used except where otherwise noted. Quality control was performed on the raw sequence reads using FastQC (Galaxy version 0.74+galaxy1) (14). Adaptors were removed via Porechop (Galaxy version 0.2.4+galaxy1); reads below 1,000 bp were filtered using Filtlong (Galaxy version 0.3.1+galaxy0) (15). After quality measures, the filtered reads were assembled on Galaxy using Flye, and a single contig, as a candidate for circularization, was confirmed by the software (Galaxy version 2.9.6+galaxy0) (16). Assembly quality was analyzed using QUAST (Galaxy version 5.3.0+galaxy1) before taxonomic classification using GTDB-Tk, requiring a minimum alignment fraction of 0.65 for alignment (Galaxy version 2.5.2+galaxy1) (17, 18). The genome was annotated using Prokka (Galaxy version 1.14.6+galaxy1) and visualized using Bakta (Galaxy version 1.9.4+galaxy1) (Fig. 1) (19, 20).

There are currently 13 published genomes of *P. lymphophilum*. ARP01 provides a second single contig assembly of a *P. lymphophilum* genome.

## Data Availability

This Whole Genome Shotgun project has been deposited at GenBank under BioProject and BioSample identifiers PRJNA1424237 and SAMN55379909, respectively. The version of the assembly described in this paper is JBVFVA010000001.1.
